# Supplementary material for: Efficacy of cochlear implants in children with borderline hearing who have already achieved significant language development with hearing aids
Source: PLoS One. 2022 Jun 1;17(6):e0267898. doi: 10.1371/journal.pone.0267898 (PMC9159549; doi:10.1371/journal.pone.0267898)
Supplement: S1 Table — Hearing preservation was calculated in subjects with functional residual hearing (pure tone thresholds ≤85dB HL at 250 and 500 Hz), RH, residual hearing. (DOCX) [file pone.0267898.s001.docx]

**S1 Table. Residual hearing preservation of pediatric cochlear implantees with borderline receptive language developments at 3 months after implantation**

| Patient No. | Implanted side | Classification of hearing preservation at postoperative 3 months |
| --- | --- | --- |
|  |  |  |
| 1 | Right | partial |
|  | Left | partial |
| 2 | Right | complete |
|  | Left | complete |
| 3 | Left | partial |
| 4 | Left | complete |
| 5 | Left | partial |
| 6 | Left | No functional RH |
| 7 | Left | minimal |
| 8 | Left | partial |
|  | Right | minimal |
| 9 | Right | partial |
| 10 | Left | minimal |
| 11 | Right | No functional RH |
| 12 | Right | No functional RH |
| 13 | Right | No functional RH |

Hearing preservation was calculated in subjects with functional residual hearing (pure tone thresholds ≤85dB HL at 250 and 500 Hz)

RH, residual hearing
