# Supplementary material for: Efficacy of cochlear implants in children with borderline hearing who have already achieved significant language development with hearing aids
Source: PLoS One. 2022 Jun 1;17(6):e0267898. doi: 10.1371/journal.pone.0267898 (PMC9159549; doi:10.1371/journal.pone.0267898)
Supplement: S2 Table — In several subjects without hearing aids used, hearing tests with rental hearing aids were performed for preoperative evaluation. Patients who underwent play audiometry due to their young age were missed at some frequencies. CI, cochlear implants; HA, hearing aids; Preop., at preoperative; Pre-HA, preoperative with hearing aids; 3M, at postoperative 3 months; 6M, at postoperative 6 months; 9-12M, at postoperative 9 to 12 months. (DOCX) [file pone.0267898.s002.docx]

S2 Table. Audiometry results at preoperative, preoperative with hearing aids and 3, 6 and 9 to 12 months after cochlear implant

| No. | Age at CI | Age at HA | HA side | CI side | Right side (250/500/1000/2000/4000Hz) | | | | |
| --- | --- | --- | --- | --- | --- | --- | --- | --- | --- |
|  |  |  |  |  | Preop. | Pre-HA | 3M  (With CI) | 6M  (With CI) | 9-12M  (With CI) |
| 1 | 54 | 50 | B | R -> L | 25/55/80/90/100 | 30/35/40/50/120 | 50/70/90/110/120  (30/30/40/35/35) | 50/70/100/120/120  (25/25/30/30/30) | 45/65/105/120/120  (30/30/30/25/25**)** |
| 2 | 63 | 12 | B | B | -/75/75/75/- | -/50/50/55/- | -/-/75/-/-  (-/45/40/40/-) | Not done  (-/35/30/35/-) | Not done |
| 3 | 67 | 52 | B | L | 40/20/25/80/85 | 40/25/15/35/80 | 50/35/10/50/60 | 85/100/110/115/120 | 100/100/105/115/120 |
| 4 | 70 | 62 | B | L | 10/30/65/70/70 | 15/25/35/45/55 | 15/40/80/85/90 | 20/35/80/85/85 | 25/40/80/90/90 |
| 5 | 94 | 23 | R | L | 70/80/80/75/75 | 30/35/35/30/30 | 65/75/75/75/75 | 70/75/75/75/70 | 70/75/75/75/75 |
| 6 | 71 | 70 | B | L | 20/40/50/50/60 | Not done | 45/45/40/40/70 | 50/45/40/45/75 | 45/45/40/35/65 |
| 7 | 74 | 44 | B | L | 60/70/75/65/60 | 50/50/45/40/35 | 70/80/85/70/70 | 75/85/85/70/70 | 75/90/90/75/65 |
| 8 | 115 | 108 | B | L -> R | 55/65/75/80/90 | 25/30/35/35/45 | 60/75/80/85/95 | 90/110/115/115/120 | 75/100/100/110/120 |
| 9 | 45 | 6 | L | R | 50/80/100/105/105 | 55/55/60/70/85 | 85/95/110/115/120  (35/35/30/30/30) | 80/90/110/115/115  (35/35/35/35/35) | 85/90/110/110/120  (30/25/25/30/30) |
| 10 | 67 | 48 | B | L | 20/30/35/35/45 | 10/20/30/25/25 | 10/25/30/35/40 | 15/25/35/30/40 | 15/30/40/35/45 |
| 11 | 48 | 12 | L | R | -/90/90/105/105 | Not done | -/-/100/-/-  (-/-/30/-/-) | -/-/100/-/-  (-/-/30/-/-) | -/-/105/-/-  (-/-/35/-/-) |
| 12 | 26 | - | - | R | -/-/100/-/- | -/-/80/-/- | -/-/100/-/-  (-/-/70/-/-) | -/-/100/-/-  (-/-/35/-/-) | -/100/100/95/-  (-/30/20/30/-) |
| 13 | 26 | 13 | B | R | -/-/70/-/- | -/-/40/-/- | Not done | -/90/105/105/120  (-/25/35/35/35) | 85/100/110/105/115  (25/25/25/30/25) |

| No. | Age at CI | Age at HA | HA side | CI side | Left side (250/500/1000/2000/4000Hz) | | | | |
| --- | --- | --- | --- | --- | --- | --- | --- | --- | --- |
|  |  |  |  |  | Preop. | Pre-HA | 3M | 6M | 9-12M |
| 1 | 54 | 50 | B | R -> L | 30/55/80/90/115 | 25/30/40/50/60 | 40/65/85/95/120 | 60/80/105/120/120 | 55/75/110/120/120 |
| 2 | 63 | 12 | B | B | -/70/70/75/- | -/45/45/55/- | -/40/40/35/- | Not done  (-/40/35/35/-) | Not done |
| 3 | 67 | 52 | B | L | 50/50/55/95/115 | 35/25/30/55/110 | 75/70/70/70/70  (45/40/25/20/35) | 85/100/110/115/120  (40/30/25/40/35) | 95/100/105/115/120  (20/30/25/25/25) |
| 4 | 70 | 62 | B | L | 15/35/70/70/70 | 15/25/35/55/65 | 40/55/85/90/85  (25/35/35/30/75) | 40/50/85/85/80  (35/35/30/30/40) | 35/50/85/85/80  (30/30/30/30/30) |
| 5 | 94 | 23 | R | L | 65/85/85/90/90 | 30/40/40/45/45 | 85/90/90/85/90  (20/25/20/15/25) | 85/105/100/90/85  (20/15/25/25/20) | 100/110/110/100/100  (20/25/20/20/20) |
| 6 | 71 | 70 | B | L | 65/100/120/120/120 | Not done | 100/115/120/110/120  (35/35/40/30/35) | 100/115/120/115/120  (35/35/40/30/35) | 110/120/120/115/120  (35/30/35/30/30) |
| 7 | 74 | 44 | B | L | 60/65/75/60/60 | 50/50/45/40/40 | 95/110/115/115/110  (25/35/25/30/40) | 110/120/120/110/120  (25/35/20/25/25) | 105/115/120/115/120  (25/30/25/25/25) |
| 8 | 115 | 108 | B | L -> R | 60/70/105/115/120 | 25/25/45/65/80 | 75/90/120/115/120  (25/25/25/20/25) | 80/100/120/115/120  (20/25/25/20/25) | 80/100/120/120/120  (20/25/25/25/25) |
| 9 | 45 | 6 | L | R | 20/65/90/90/95 | 30/45/40/50/60 | 45/70/85/85/90 | 50/55/45/55/65 | 45/60/85/90/105 |
| 10 | 67 | 48 | B | L | 50/85/90/85/120 | 25/50/50/45/50 | 90/110/120/115/120  (25/30/25/25/30) | 95/110/120/115/120  (15/20/20/25/25) | 105/115/120/120/120  (35/35/30/30/30) |
| 11 | 48 | 12 | L | R | -/70/70/60/70 | -/50/45/35/45 | -/-/45/-/- | -/-/45/-/- | -/-/35/-/- |
| 12 | 26 | - | - | R | -/-/10/-/- | -/-/10/-/- | -/-/15/-/- | -/-/20/-/- | -/15/15/15/- |
| 13 | 26 | 13 | B | R | -/-/40/-/- | -/-/25/-/- | Not done | -/35/35/35/50 | 50/60/60/60/70 |

In several subjects without hearing aids used, hearing tests with rental hearing aids were performed for preoperative evaluation. Patients who underwent play audiometry due to their young age were missed at some frequencies. CI, cochlear implants; HA, hearing aids; Preop., at preoperative; Pre-HA, preoperative with hearing aids; 3M, at postoperative 3 months; 6M, at postoperative 6 months; 9-12M, at postoperative 9 to 12 months
