# Supplementary material for: Efficacy of cochlear implants in children with borderline hearing who have already achieved significant language development with hearing aids
Source: PLoS One. 2022 Jun 1;17(6):e0267898. doi: 10.1371/journal.pone.0267898 (PMC9159549; doi:10.1371/journal.pone.0267898)
Supplement: S3 Table — Het, heterozygote; Homo, homozygote; VUS, Variant of Uncertain Significance; NA, not applicable; ND, no data. (DOCX) [file pone.0267898.s003.docx]

**S3 Table. Details of genetypes of pediatric cochlear implantees with borderline receptive language developments before surgery**

| Patient No. | Gene  [Reference Sequences] | Variant | | Zygosity | Insilico Predictions | | Alternative Allele Frequency | | ACMG-AMP evidence of Pathogenicity | |  |
| --- | --- | --- | --- | --- | --- | --- | --- | --- | --- | --- | --- |
|  |  |  |  |  | CADD Phred | REVEL | KRGDB  (1722 individuals) | GMAF | Criteria | Classification |  |
| 1 | USH2A  [NM_206933.2]  [NP_996816.2] | c.15233C>G:p.Pro5078Arg | | Het | 27.20 | 0.432 | C=0.004936/17 | C=0.000007  (1/140218, GnomAD) | PM2, PP4 | VUS |  |
| 2 | PDZD7  [NM_001195263.1]  [NP_001182192.1] | c.490C>T:p.Arg164Trp | | Het | 24.30 | 0.377 | A=0.0029/10 | A=0.00005/6 (ExAC)  A=0.0002/1 (1000 Genomes) | PM2, PM3_VS | Likely Pathogenic |  |
|  |  | c.1669del:p.Arg557Gly*fs**13 | | Het | NA | NA | ND | ND | PVS1, PM2, PM3_P | Pathogenic |  |
| 3 | SLC26A4  [NM_000441.2]  [NP_000432.1] | c.1229C>T:p.Thr410Met | | Het | 25.20 | 0.907 | ND | T=0.000043  (6/140200, GnomAD) | PM3_VS, PP1_S, PM2, PP3, PS3_P, PP4 | Pathogenic |  |
|  |  | c.2027T>A:p.Leu676Gln | | Het | 26.30 | 0.733 | A=0.000874/3 | A=0.000015  (4/264690, TOPMED) | PM2, PM3_VS, PP3, PP4 | Pathogenic |  |
| 4 | MYO15A  [NM_016239.3]  [NP_057323.3] | c.419del:p.Lys140Ser*fs**304 | | Het | NA | NA | ND | delA=0.000009  (1/111296, ExAC) | PVS1, PM2 | Likely Pathogenic |  |
|  |  | c.9478C>T:p.Leu3160Phe | | Het | 23.00 | 0.729 | T=0.005824/20 | T=0.006581  (923/140252, GnomAD) | PM3_P, PP3 | VUS |  |
| 5 | Congenital CMV infection | | |  |  |  |  |  |  |  |  |
| 6 | Congenital CMV infection | | |  |  |  |  |  |  |  |  |
| 7 | MYO6  [NM_004999.3]  [NP_004990.3] | c.667G>A:p.Gly223Arg | | Het | 29.80 | 0.923 | ND | ND | PS4_P, PM2, PP1_M, PP3 | VUS |  |
| 8 | Unknown |  | |  |  |  |  |  |  |  |  |
| 9 | SLC26A4  [NM_000441.2]  [NP_000432.1] | c.2168A>G:p.His723Arg | | Homo | 25.20 | 0.933 | G=0.005824/20 | G=0.000036  (5/140300, GnomAD) | PM2, PM3_VS, PP3, PP4 | Pathogenic |  |
| 10 | NLRP3  [NM_001243133.1]  [NP_001230062.1] | c.2062G>A:p.Glu688Lys | | Het | 21.80 | 0.494 | ND | ND | PS2, PM2 | Likely Pathogenic |  |
| 11 | Congenital CMV infection | | |  |  |  |  |  |  |  |  |
| 12 | Congenital CMV infection | | |  |  |  |  |  |  |  |  |
| 13 | SLC26A4  [NM_000441.2]  [NP_000432.1] | | c.919-2A>G | Homo | 34.00 | NA | G=0.000873/3 | G=0.000178  (25/140276, GnomAD) | PVS1, PP1_S, PM3_VS, PP3, PP4 | Pathogenic | |

Het, heterozygote; Homo, homozygote; VUS, Variant of Uncertain Significance; NA, not applicable; ND, no data

Combined Annotation Dependent Depletion (CADD) <https://cadd.gs.washington.edu/>

Rare Exome Variant Ensemble Learner (REVEL) https://sites.google.com/site/revelgenomics/

KRGDB <http://152.99.75.168:9090/KRGDB/welcome.jsp>
