# Supplementary material for: Efficacy of cochlear implants in children with borderline hearing who have already achieved significant language development with hearing aids
Source: PLoS One. 2022 Jun 1;17(6):e0267898. doi: 10.1371/journal.pone.0267898 (PMC9159549; doi:10.1371/journal.pone.0267898)
Supplement: S4 Table — U-TAP, Urimal Test of Articulation and Phonation; SD, standard deviation; K-CID, Korean version of the Central Institute for the Deaf; Preop., at preoperative; 3M, at postoperative 3 months; 6M, at postoperative 6 months; 9-12M, at postoperative 9 to 12 months; UC, uncheckable. (DOCX) [file pone.0267898.s004.docx]

S4 Table. Speech evaluation results at preoperative and 3, 6 and 9 to 12 months after cochlear implant

| No. |  | CAP score | | | |  | IT-MAIS | | | |  | SIR | | | |
| --- | --- | --- | --- | --- | --- | --- | --- | --- | --- | --- | --- | --- | --- | --- | --- |
|  |  | Preop. | 3M | 6M | 9-12M |  | Preop. | 3M | 6M | 9-12M |  | Preop. | 3M | 6M | 9-12M |
| 1 |  | 5 | 7 | 7 | 7 |  | 34 | 40 | 40 | 39 |  | 2 | 3 | 3 | 4 |
| 2 |  | 5 | 5 | 5 | 5 |  | 39 | 40 | 40 | 40 |  | 3 | 3 | 3 | 4 |
| 3 |  | 6 | 7 | 7 | 7 |  | 39 | 39 | 39 | 40 |  | 5 | 5 | 5 | 5 |
| 4 |  | 6 | 6 | 6 | 6 |  | 39 | 40 | 40 | 40 |  | 4 | 4 | 4 | 5 |
| 5 |  | 6 | 6 | 6 | 6 |  | 38 | 34 | 39 | 40 |  | 5 | 5 | 5 | 5 |
| 6 |  | 6 | 7 | 7 | 7 |  | 39 | 39 | 40 | 40 |  | 4 | 4 | 5 | 5 |
| 7 |  | 6 | 6 | 6 | 7 |  | 39 | 40 | 39 | 40 |  | 5 | 5 | 5 | 5 |
| 8 |  | 6 | 7 | 7 | 7 |  | 38 | 39 | 39 | 40 |  | 5 | 5 | 5 | 5 |
| 9 |  | 6 | 6 | 6 | 6 |  | 38 | 39 | 40 | 40 |  | 3 | 3 | 4 | 4 |
| Mean  ±SD |  | 5.8±0.4 | 6.3±0.7 | 6.3±0.7 | 6.4±0.7 |  | 38.1±1.5 | 38.9±1.8 | 39.6±0.5 | 39.9±0.3 |  | 4±1.1 | 4.1±0.9 | 4.3±0.8 | 4.7±0.5 |
| 10 |  | 6 | 6 | 6 | 7 |  | 31 | 38 | 38 | 38 |  | 4 | 4 | 4 | 5 |
| 11 |  | 5 | 5 | 5 | 5 |  | 35 | 32 | 32 | 35 |  | 1 | 1 | 1 | 1 |
| 12 |  | 5 | 5 | 6 | 6 |  | UC | 32 | 39 | - |  | 2 | 4 | 4 | - |
| 13 |  | 5 | - | - | 5 |  | 30 | - | - | - |  | 2 | - | - | - |

| No. |  | U-TAP score (%) | | | |  | Word perception (%) | | | |  | K-CID (%) | | | |
| --- | --- | --- | --- | --- | --- | --- | --- | --- | --- | --- | --- | --- | --- | --- | --- |
|  |  | Preop. | 3M | 6M | 9-12M |  | Preop. | 3M | 6M | 9-12M |  | Preop. | 3M | 6M | 9-12M |
| 1 |  | 60.46 | 75.58 | 90.69 | 90.69 |  | 15 | 52.5 | 90 | 95 |  | 100 | 100 | 100 | 100 |
| 2 |  | 44.18 | 46.51 | 46.51 | 60.46 |  | UC | 50 | 70 | 55 |  | UC | UC | UC | UC |
| 3 |  | 88.37 | 97.67 | 97.67 | 100 |  | 75 | 85 | 90 | 95 |  | 100 | 99.5 | 100 | 100 |
| 4 |  | 79.06 | 81.39 | 88.37 | 95.34 |  | 80 | 50 | 80 | 90 |  | 90 | 90 | 100 | 100 |
| 5 |  | 93.02 | 97.67 | 97.67 | 97.67 |  | 80 | 75 | 100 | 100 |  | 96.6 | 96.6 | 100 | 100 |
| 6 |  | 81.39 | 86.04 | 90.69 | 97.67 |  | 80 | 85 | 90 | 85 |  | 96.6 | 96.6 | 100 | 100 |
| 7 |  | 97.67 | 97.67 | 97.67 | 100 |  | 65 | 75 | 90 | 95 |  | 100 | 100 | 100 | 100 |
| 8 |  | 97.67 | 97.67 | 97.67 | 100 |  | 61 | 90 | 90 | 95 |  | 86 | 100 | 100 | 100 |
| 9 |  | 76.74 | 72.09 | 86.04 | 90.69 |  | 35 | 40 | 85 | 80 |  | 93.3 | 96.6 | 100 | 100 |
| Mean  ±SD |  | 80.81  ±13.09 | 79.84  ±16.81 | 88.11  ±15.33 | 92.50  ±11.85 |  | 61.4  ±22.6 | 66.9  ±17.7 | 87.2  ±7.9 | 87.8  ±12.9 |  | 95.3  ±4.9 | 97.4  ±3.2 | 100 | 100 |
| 10 |  | 86.04 | 90.69 | 90.69 | 100 |  | 35 | 90 | 75 | 90 |  | 100 | 96.6 | 96.6 | 100 |
| 11 |  | UC | UC | UC | UC |  | UC | UC | UC | UC |  | UC | UC | UC | UC |
| 12 |  | UC | 83.72 | 86.04 | 81.39 |  | UC | 85 | 100 | 100 |  | UC | UC | 100 | 100 |
| 13 |  | UC | - | - | 37.21 |  | UC | - | - | 56 |  | UC | - | - | 67 |

U-TAP, Urimal Test of Articulation and Phonation; SD, standard deviation; K-CID, Korean version of the Central Institute for the Deaf; Preop., at preoperative; 3M, at postoperative 3 months; 6M, at postoperative 6 months; 9-12M, at postoperative 9 to 12 months; UC, uncheckable
