# Supplementary material for: Efficacy of cochlear implants in children with borderline hearing who have already achieved significant language development with hearing aids
Source: PLoS One. 2022 Jun 1;17(6):e0267898. doi: 10.1371/journal.pone.0267898 (PMC9159549; doi:10.1371/journal.pone.0267898)
Supplement: Attachment — Submitted filename: rebuttal letter.docx [file pone.0267898.s005.docx]

Reviewer #1: This manuscript deals with the postoperative outcome for those with limited progress made with conventional hearing aid use with cochlear implantation. As the authors pointed out, there are uncertainties regarding the point of surgical intervention using cochlear implantation especially when a child has residual hearing. Thus, the authors are addressing clinically relevant and important questions. Although the stated questions are valid, there are some areas that require further clarification.

There are total of 13 patients who were selected for this current study but it is unclear as to when the patients started using hearing aids for the first time, and how long the hearing aids were used prior to surgery. It appears that some of the patients in the study did not have any usage of hearing aid. Patient 2 can be defined as a case of delayed intervention with bilateral congenital profound hearing loss without any prior use of hearing aids. By reviewing the audiometric criteria presented for the 13 patients, some of the patients can be characterized as receiving cochlear implantation for single sided deafness. Unless the patient selection criteria is better defined, there are some selection bias. The duration of deafness, clear audiologic definition for surgical intervention, methods of preoperative and postoperative rehabilitations are not available. Thus, the authors are encouraged to present detailed clinical information for each patient. In this study, the definition of functional residual hearing threshold was defined as 85dB but, it is highly questionable that a patient can obtain speech discrimination at this level using a hearing aid. Therefore, the definition of functional hearing preservation needs better definition.

Thank you for the opportunity to supplement the important points. We added the data of timing and sides of hearing aids and preoperative and postoperative audiometry of each patients in supplementary table S2. In these borderline cases, we decided cochlear implantation not by audiologic definition but by consultation with the guardian in consideration of language development including pronunciation accuracy.

The reason we dealt with the preservation of residual hearing in this study was to clarify the safety of the surgery in these subjects. So pure tone thresholds ≤85dB HL at 250 and 500 Hz are the thresholds used as a standard for measuring rates of preserving residual hearing in cochlear implant surgery suggested by HEARRING group. To make the point clear, we edited the manuscript. Please refer line 75-78 and 285-288 of the manuscript.

In the supplement material provided, the hearing preservation data is presented only at three months following surgery only qualitative data is presented. Since these patients were followed for 12 months following surgery, it is recommended to present the actual audiometric data for these nine patients. Since the number of patients in the current study is rather small, the authors are encouraged to present all of the longitudinal audiologic and speech perception data in the first 12 months period at the individual level. Once all of the audiologic data and clinical data are available for all of these patients, authors should also look into the difference of outcome using different cochlear implant electrodes.

Thanks for the good point. Audiologic and speech evaluation data including CAP, IT-MAIS and SIR scores for 12 months in each subjects were added in the supplementary material. As shown in Table 1 of main manuscript, CI 522, 532 and 632 electrodes were used. Because of small number of subjects, we could not study the difference between different electrodes.

For the revision, it is recommended to shorten the manuscript since there are some areas that are redundant.

Thanks for the good comment. The manuscript has been edited to be more concise.
